# Outcomes of Aquablation in BPH with bladder stones: Analysis of the ICARUS database

**DOI:** 10.1002/bco2.70156

**Published:** 2026-01-30

**Authors:** Joshua D. Cabral, Margaret Gannon, Gregory Raster, David J. Nusbaum, David Bouhadana, Adel Arezki, Aalya Hamouda, Anouk Leathead, Ilan Ohana, Rosie Foucault, Iman Sadri, Jeffrey A. Sioufi, Nick Lee, Tarek Benzouak, Liam Murad, Nicholas J. Corsi, John Klein, Inderjit Singh, James Kearns, Cecilia Chang, Juan Justo Quintas, Kevin C. Zorn, Tiago Rodrigues, Shawn H. Marhamati, Brian T. Helfand, Alexander P. Glaser

**Affiliations:** ^1^ Department of Surgery, Division of Urology University of Chicago Chicago Illinois USA; ^2^ Department of Surgery, Division of Urology McGill University Montreal Quebec Canada; ^3^ Department of Surgery, Division of Urology University of Montreal Montreal Quebec Canada; ^4^ Department of Surgery, Division of Urology University Laval Quebec City Quebec Canada; ^5^ Department of Surgery, Division of Urology University of Sherbrooke Sherbrooke Quebec Canada; ^6^ Department of Urology University of Texas Southwestern Medical Center Dallas Texas USA; ^7^ Potomac Urology Center National Harbor Maryland USA; ^8^ Department of Surgery, Division of Urology Endeavor Health Evanston Illinois USA; ^9^ ROC Clinic Madrid Spain; ^10^ Department of Urology HM Hospitales Madrid Spain; ^11^ HM Hospitals Faculty of Health Sciences Camilo José Cela University Madrid Spain; ^12^ BPH Canada Prostate Surgical Institute Montreal Quebec Canada; ^13^ Department of Surgery, Division of Urology Hospital Cruz Vermelha Lisbon Portugal

**Keywords:** Aquablation, bladder stones, BPH, cystolitholapaxy, Medicare

## Abstract

**Objective:**

The purpose of this study is investigate the clinical outcomes of men with benign prostatic hyperplasia (BPH) and bladder stones treated concomitantly with Aquablation and bladder stone removal in an international, multi‐institutional cohort.

**Patients and Methods:**

We performed a retrospective analysis of men who underwent Aquablation between 2018 and 2024. Patients were divided into two cohorts: men with bladder stones and those without. Outcomes assessed included baseline demographics and variables (medication use, prostate volume, prior BPH surgery), operative characteristics (OR time, transfusion requirement, complications), and functional outcomes were measured by the International Prostate Symptom Score (IPSS), peak urinary flow rate (*Q*_max_) and PVR at regular intervals over 24 months.

**Results:**

A total of 1885 men were analysed, including 60 men with bladder stones and 1825 without. Patients with bladder stones had higher rates of prior BPH surgery (15% vs. 5.5%, *p* = 0.015) and pre‐operative urinary retention (28.3% vs. 14.8%, *p* = 0.004) as well as larger prostate volumes (98.6 ml vs. 77 ml, *p* < 0.0001). Total operative time was longer in the bladder stone group (76.5 mins vs. 55.0 mins, *p* < 0.001), but there were no significant differences in Aquablation time or bleeding complications including transfusions. There was also no difference in improvements in IPSS, *Q*_max_ and PVR post‐operatively.

**Conclusion:**

This study demonstrates that Aquablation is safe and effective in the management of BPH regardless of concomitant bladder stone treatment.

## 1 INTRODUCTION

Benign prostatic hyperplasia (BPH) is a common condition among ageing men which consequently can result in bladder outlet obstruction (BOO) and subsequent lower urinary tract symptoms (LUTS).^1^, ^2^, ^3^ The prevalence of LUTS/BPH increases with age with approximately 80% of men aged 70 years or older being affected.^4^, ^5^ With disease progression, worsening symptoms can have a profound impact on quality of life (QoL), mental health and health‐care expenditure.^6^, ^7^, ^8^, ^9^, ^10^ These negative impacts can be exacerbated by additional complications of BOO, such as urinary retention, renal insufficiency, recurrent urinary tract infections and/or bladder stones. Bladder stones most often result from urinary stasis secondary to BOO and incomplete voiding.^11^ The presence of these stones can be associated with urinary infections, irritative voiding symptoms, haematuria and pain.^12^ In addition, bladder stones may be a harbinger for severe BOO, and it is unknown if surgical relief of BPH is always associated with similar clinical outcomes to men without stones. Bladder stones are typically managed with endoscopic cystolitholapaxy, often using holmium: YAG or thulium fibre laser fragmentation along with concomitant BPH surgery as men who do not undergo bladder stone removal are at much higher risk of recurrent bladder stones and subsequent BPH surgeries.^13^ This is reflected in current American Urological Association (AUA) guidelines that recommend surgical treatment of BPH in men with recurrent bladder stones.^14^

Aquablation (PROCEPT BioRobotics) is an image‐guided, robotically executed surgical procedure utilising high‐velocity water jet technology and has emerged as an effective treatment for BPH. Pivotal randomised controlled trials have demonstrated that Aquablation is both a safe and effective therapy with durable outcomes, significant improvement in functional outcomes and preservation of sexual function.^15^, ^16^, ^17^, ^18^, ^19^, ^20^, ^21^

Despite this growing evidence base, there is little published regarding its use in men with concomitant bladder stones, a subgroup that may present distinct perioperative and functional considerations. In part, because of this lack of evidence, Medicare coverage determinations currently restrict the use of Aquablation in the presence of bladder stones, thus limiting utilisation of Aquablation in otherwise ideal patients who desire BPH treatment with Aquablation.^22^ This study aimed to investigate the efficacy, safety and clinical outcomes of Aquablation in men with BPH and concurrent bladder stones in a large multi‐institutional retrospective cohort.

## 2 METHODS

### 2.1 Study design and population

This retrospective cohort study analysed men who underwent Aquablation for BPH between 2018 and 2024 at one of four sites in the International Collaborative Aquablation Research Urology Society (ICARUS) database. Male patients diagnosed with symptomatic LUTS/BPH who underwent Aquablation were included in the study. Study participants were divided into two cohorts: men with bladder stones undergoing concomitant cystolitholapaxy (stone group) and those without (non‐stone group). Patients with unknown or unspecified bladder stone status were excluded from this analysis (*N* = 259). This study was approved by the institutional review board (IRB; EH20‐122).

### 2.2 Clinical variables and outcomes

Baseline data collected included demographic information (age, comorbidities), pre‐operative medication use (e.g. alpha‐blockers, 5‐alpha‐reductase inhibitors, daily phosphodiesterase 5 inhibitors, anticoagulation), serum prostate specific antigen (PSA) values, serum prostate health index (PHI) values, prostate volume and history of prior BPH surgeries. Pre‐operative characteristics included maximum urinary flow rate (*Q*_max_), post‐void residual volume (PVR) and patient‐related outcomes, including the International Prostate Symptom Score (IPSS), IPSS quality of life (QoL) and Sexual Health Inventory for Men (SHIM).

Study outcomes were assessed at baseline and at regular intervals post‐operatively. Functional outcomes were assessed using the IPSS, SHIM, uroflowmetry and PVR. Additionally, operative and peri‐operative outcomes were collected including total operative time, total Aquablation time, transfusion requirements, takebacks for cystoscopy with clot evacuation and other surgical complications as classified on the Clavien–Dindo scale. Additionally, stone characteristics such as stone size, number of stones, stone diameter, method of removal and stone composition were recorded. Systematic use of the Male Sexual Health Questionnaire assessing Ejaculatory Dysfunction (MSHQ–EjD) was limited in the ICARUS database; therefore, ejaculatory function was also categorically recorded by sites as normal antegrade ejaculation, antegrade ejaculation with reduced volume, and retrograde ejaculation.

### 2.3 Statistical analysis

Statistical analyses were performed to compare the characteristics between men with and without bladder stones. Continuous variables were expressed as means with standard deviations (SD) or medians with interquartile ranges (IQR), as appropriate. Comparisons between groups were performed using *t*‐tests or Mann–Whitney *U* tests for continuous variables and chi‐square tests for categorical variables. Missing data were handled with pairwise deletion. Statistical significance was defined as a *p*‐value < 0.05. Statistical analysis was performed with SAS v9.4 (SAS Institute, Cary, NC) and R v4.3.2.

## 3 RESULTS

### 3.1 Patient characteristics

The analysis included 1885 men who underwent Aquablation from 2018 to 2024. Of those men, 60 (3.2%) patients had bladder stones and 1825 did not. Table 1 displays the baseline characteristics of the two groups.

**TABLE 1:** Demographics and baseline variables.

	No bladder stones (*N* = 1825)		Men with bladder stones (*N* = 60)		*p*‐value
	*N*		*N*		
Age, years (mean, SD)	1825	68.98 (8.03)	60	70.75 (7.57)	0.09
Diabetes mellitus type 2 (*n*, %)	579	90 (15.54)	38	8 (21.05)	0.37
Coronary artery disease (*n*, %)	261	36 (13.79)	16	4 (25.00)	0.26
Anxiety/depression (*n*, %)	579	102 (17.62)	36	5 (13.89)	0.57
Obstructive sleep apnoea (*n*, %)	479	111 (23.17)	31	8 (25.81)	0.74
Family history of BPH (*n*, %)	375	116 (30.93)	22	5 (22.73)	0.42
HbA1c (median, IQR)	213	5.60 (5.40–6.00)	11	5.70 (5.40–6.10)	0.64
PSA (median, IQR)	1447	3.65 (1.91–6.46)	55	4.55 (2.86–6.93)	0.05
Prostate Health Index (median, IQR)	405	30.79 (23.59–41.09)	28	32.26 (26.01–44.92)	0.33
Baseline medication use (*n*, %)					
Alpha adrenergic antagonist	732	536 (73.22)	48	32 (66.67)	0.32
5ARI	732	147 (20.08)	48	10 (20.83)	0.90
Daily PDE5‐i	640	94 (14.69)	43	8 (18.60)	0.49
Anticholinergic or β3 agonist	732	34 (4.64)	48	2 (4.17)	1.00
Prostate volume, cc (median, IQR)	1809	77.00 (55.50–107.65)	60	98.57 (79.00–141.50)	<0.0001
Median lobe (*n*, %)	1688	1122 (66.47)	53	33 (62.26)	0.52
Intravesical prostatic protrusion (*n*, %)					
Small (<5 mm)	592	138 (23.31)	23	7 (30.43)	0.30
Moderate (5–10)		102 (17.23)		6 (26.09)	
Large (>10 mm)		352 (59.46)		10 (43.48)	
Anticoagulation, any (*n*, %)	661	175 (26.48)	45	14 (31.11)	0.50
Aspirin 81 mg (*n*, %)	490	75 (15.31)	35	8 (22.86)	0.24
Other antiplatelet agent (e.g. clopidogrel, prasugrel) (*n*, %)	490	19 (3.88)	35	0 (0.00)	0.63
Direct oral anticoagulant or warfarin (*n*, %)	490	56 (11.43)	35	6 (17.14)	0.28
Prior BPH surgery (*n*, %)					
No	1821	1721 (94.51)	60	51 (85.00)	0.015
Prostate artery embolisation		6 (0.33)		0 (0.00)	
Photoevaporation of prostate		19 (1.04)		3 (5.00)	
Robotic simple prostatectomy		3 (0.16)		0 (0.00)	
Water vapour thermotherapy (Rezum)		23 (1.26)		0 (0.00)	
Transurethral incision of prostate (TUIP)		1 (0.05)		0 (0.00)	
Transurethral microwave thermotherapy (TUMT)		2 (0.11)		0 (0.00)	
Transurethral resection of prostate (TURP)		20 (1.10)		2 (3.33)	
Prostatic urethral lift (Urolift)		25 (1.37)		4 (6.67)	
Robotic waterjet therapy (Aquablation)		1 (0.05)		0 (0.00)	
*Q*_max_, ml/s (median, IQR)	911	8.50 (5.40–12.60)	18	6.00 (5.00–8.80)	0.06
PVR, ml (median, IQR)	1351	106.00 (41.00–219.00)	50	112.00 (38.00–321.00)	0.38
Urinary retention requiring catheter or CIC (*n*, %)	1822	269 (14.76)	60	17 (28.33)	0.004
Baseline PROs					
IPSS (mean, SD)	1594	20.40 (7.65)	51	19.43 (7.88)	0.37
QOL (median, IQR)	546	4.00 (3.00–5.00)	37	4.00 (3.00–5.00)	0.50
SHIM (mean, SD)	886	14.33 (8.15)	38	14.21 (8.31)	0.93

Patients with bladder stones had significantly larger prostate volumes (99 ml vs. 77 ml, *p* < 0.0001), were more likely to have previously undergone BPH surgery (15% vs. 5.5%, *p* = 0.015) and were more likely to be in pre‐operative urinary retention (28.3% vs. 14.8%, *p* = 0.004). Men with bladder stones also tended to have higher serum PSA values compared to those without stones (median PSA 4.55 vs. 3.65 ng/ml, *p* = 0.05). Baseline urinary symptoms and erectile function were similar between groups (Table 1).

### 3.2 Operative outcomes

Table 2 demonstrates the comparison of intra‐operative details between the two groups. As expected, the group that underwent concomitant bladder stone treatment had a longer total operative time (76 vs. 55 min, *p* < 0.0001). However, the two groups had no significant differences in the Aquablation‐specific BPH treatment time, transfusion rates, complications or 30‐day readmissions.

**TABLE 2:** Operative characteristics.

	No bladder stones (*N* = 1825)		Men with bladder stones (*N* = 60)		*p*‐value
Total OR time, min, (median, IQR)	1784	55.00 (43.00–70.00)	60	76.50 (58.50–108.00)	<0.0001
Aquablation time, min, (median, IQR)	538	6.98 (5.20–8.47)	35	7.03 (6.27–8.68)	0.24
Takeback for cystoscopy/clot evacuation (*n*, %)	1825	38 (2.08)	60	1 (1.67)	1.00
Blood transfusion (*n*, %)	1825	12 (0.66)	60	2 (3.33)	0.07
30‐day readmission (*n*, %)	1510	16 (1.06)	42	0 (0.00)	1.00
Clavien–Dindo complications (*n*, %)					
Clavien 0	1735	1517 (87.44)	51	42 (82.35)	0.11
Clavien I		148 (8.53)		4 (7.84)	
Clavien II		19 (1.10)		3 (5.88)	
Clavien IIIa		6 (0.35)		0 (0.00)	
Clavien IIIb		44 (2.54)		2 (3.92)	
Clavien V		1 (0.06)		0 (0.00)	
Stone characteristics					
Stone size, mm (median, IQR)		/		10 (5–20)	
Number of bladder stones (median, IQR)		/		1.5 (1–4)	
Aggregate stone diameter, mm × N, (median, IQR)		/		22.5 (10.0–38.8)	
Stones treated with laser lithotripsy, %		/		70% (*N* = 42)	
Stones treated with extraction/removal, %		/		30% (*N* = 18)	
30‐day UTI rate				5%	

### 3.3 Functional outcomes

**FIGURE 1:**
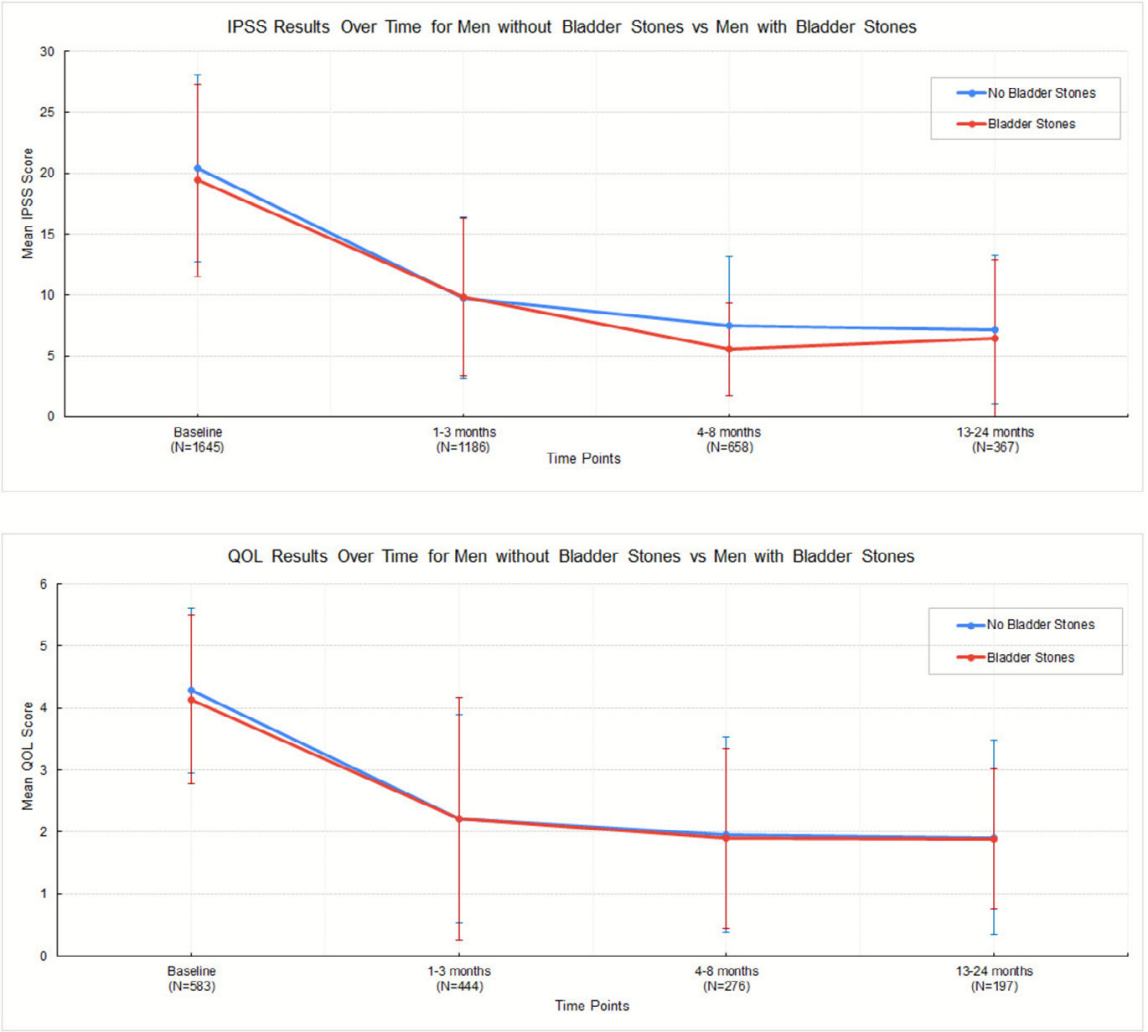
Improvement in IPSS and IPSS QoL after Aquablation in men with and without concomitant treatment of bladder stones. IPSS, International Prostate Symptom Score; IPSS QoL, International Prostate Symptom Score quality of life.

Over the 24‐month follow‐up period, both groups displayed similar and notable improvements in IPSS (Figure 1). IPSS improved similarly from baseline 19.4 vs. 20.4 (*p* = 0.37) to 9.85 vs. 9.74 (*p* = 0.91) at 3 months post‐operatively, and 6.46 vs. 7.13 (*p* = 0.70) at 24 months in the bladder stone and non‐stone groups, respectively. There was a significant difference in IPSS measured 4–8 months post‐operatively (5.54 ± 3.81 [*n* = 26, bladder stones] vs. 7.48 ± 5.72 [*n* = 632, no bladder stones], *p* = 0.004), but there were no other significant differences at any other timepoints. Both groups also reported similar improvements in IPSS and QoL scores over the follow‐up period.

Both groups also had similar post‐operative *Q*_max_ (19.3 vs. 19.5, p = 0.78) and post‐operative PVR (24 ml vs 18 ml, *p* = 0.18) (Table 3). Post‐operative PSA was slightly higher in the bladder stone group (3.32 vs 2.05, *p* = 0.017), but there was no difference in change in PSA from baseline, percent change in PSA from baseline, or in post‐operative Prostate Health Index between groups. Furthermore, no differences were observed in post‐operative SHIM scores between groups, and absolute changes from baseline were similar. Also of importance, there was no difference in the rate of men who reported ejaculatory dysfunction between groups.

**TABLE 3:** Post‐operative variables (data collected 1–3 months post‐op).

	No bladder stones (*N* = 1825)		Men with bladder stones (*N* = 60)		*p*‐value
Post‐op *Q*_max_, ml/s (median, IQR)	627	19.3 (14.6–26.0)	8	19.5 (16.8–24.6)	0.78
Post‐op change in *Q*_max_ from baseline, ml/s (median, IQR)	474	10.9 (6.2–17.9)	8	12.35 (10.9–17.2)	0.41
% change in *Q*_max_	474	142% (62%–278%)	8	245% (179%–329%)	0.13
Postop PVR, ml (median, IQR)	1491	24 (6–65)	50	18 (3–50)	0.18
Postop change in PVR, ml (median, IQR)	1207	−145.57 (236.56)	44	−178.75 (282.79)	0.25
% change in PVR	1165	−80% (−97–49%)	44	−89% (−99%–40%	0.31
Post‐op SHIM (mean, SD)	650	13.43 (8.79)	34	13.65 (9.63)	0.89
Post‐op change in SHIM from baseline	543	−0.10 (5.27)	29	−1.72 (4.60)	0.10
% change in SHIM	540	−24.50 (164.24)	29	−15.21 (35.09)	<0.0001
Post‐op retrograde ejaculation (*n*, %)					
No	514	415 (80.74%)	18	17 (94.44%)	0.52
Reduced or decreased ejaculate		46 (8.95%)		0 (0.00%)	
Yes		53 (10.31%)		1 (5.56%)	
Postop PSA, ng/ml (median, IQR)	636	2.05 (1–3.56)	19	3.320 (1.87–4.57)	0.017
Postop change in PSA from baseline (median, IQR)	571	−1.60 (−3.70–0.37)	19	−2.38 (−4.12–0.95)	0.15
% change in PSA	571	−44% (−69–16%)	19	−42% (−63–19%)	0.81
Postop Prostate Health Index (median, IQR)	267	25.96 (18.80–36.05)	17	24.69 (16.67–33.04)	0.54
Postop change in Prostate Health Index from baseline (median, IQR)	204	−4.04 (−11.52–1.68)	15	−3.90 (−12.83–0.91)	0.82
% change in Prostate Health Index (median, IQR)	204	−13% (−32%–7%)	15	−15% (−36–3%)	0.64

## 4 DISCUSSION

The current treatment landscape for the surgical management of BPH is changing, with newer techniques, such as Aquablation, becoming more utilised. As these procedures become more prevalent, their utilisation and application in well‐selected patients are important. Men who form bladder stones may have a higher degree or more severe and chronic form of BOO related to BPH that results in urinary stasis and stone formation.^23^ Furthermore, BOO may be associated with structural changes in the bladder which diminish detrusor function over the long term.^24^ The current study demonstrates that Aquablation is a safe and effective treatment when performed concomitantly with laser endoscopic cystolitholapaxy for the treatment of bladder stones. In this analysis, men with bladder stones who are treated with Aquablation and concomitant bladder stone removal have similar perioperative safety and functional outcomes to those without stones who undergo Aquablation.

Current Medicare local coverage determinations preclude the use of Aquablation in the presence of bladder stones, thus necessitating patients who are insured by Medicare and interested in this procedure to either explore alternative treatment options or undergo multiple anaesthetics and staged procedures. This government policy negates shared decision making and could force men with larger prostates to undergo alternative procedures, such as transurethral resection of the prostate (TURP), simple prostatectomy or holmium laser enucleation of the prostate (HoLEP), which have higher risks of sexual dysfunction and stress urinary incontinence compared to Aquablation.^25^, ^26^ In part because of this government‐imposed restriction, limited data on concomitant treatment of bladder stones with Aquablation is available. Our current study demonstrates that Aquablation is safe and effective in treating BPH in men with bladder stones and that, over a 24‐month‐follow‐up period, the presence of bladder stones did not impact long‐term functional outcomes.

Our findings are analogous to other studies on concomitant treatment of bladder stones. Romero‐Otero et al. showed that concomitant laser cystolitholapaxy at the time of HoLEP increased total operative time by an average of 17 min; however, no differences were noted in enucleation time, morcellation time or post‐operative functional outcomes.^22^ Similarly, in our study, concomitant treatment of bladder stones increased total operative time by an average of 21.5 min, but Aquablation time, overall complication rates, transfusion requirements and long‐term functional outcomes were similar between the two cohorts.

There are several limitations to this study. The retrospective nature of the study limits the ability to establish causality and may be subject to biases. Patients with bladder stones had larger prostate volumes and were more likely to have undergone prior BPH procedures. Follow‐up was not standardised and thus variable in this real‐world cohort which led to inconsistencies in cohort follow‐up for functional outcomes, and not all patients completed 24‐month follow‐up. Furthermore, the relatively small sample size of the bladder stone group (*N* = 60) may limit the statistical power to detect true differences between the groups. As in any large, real‐world, retrospective study, missing data are inevitable. Non‐random missing data could have introduced bias and may therefore limit the generalisability of our findings. Finally, 24‐month follow‐up may be insufficient to assess long‐term complications and the need for retreatment; long‐term retreatment rates are better assessed through national databases not linked to individual institutions.

## 5 CONCLUSION

In conclusion, while the presence of bladder stones can extend total procedural time, this study demonstrates that Aquablation is a safe and effective treatment option in the management of BPH including men with bladder stones. There was no increase in transfusions or perioperative complications with concomitant treatment of bladder stones, and both cohorts demonstrated durable improvements in functional outcomes over 24 months. Based on these findings, federal health‐care policies should consider eliminating restrictions on concurrent bladder stone treatment during Aquablation, aligning with existing coverage determinations for all other BPH procedures, such as prostatic urethral lift, water vapour thermotherapy, photoselective vaporisation of the prostate, TURP and laser enucleation.

## AUTHOR CONTRIBUTIONS

**Joshua D. Cabral:** Writing—original draft. **Margaret Gannon:** Writing—original draft. **Gregory Raster:** Writing—original draft. **David J. Nusbaum:** Writing—original draft. **David Bouhadana:** Writing—review and editing. **Adel Arezki:** Writing—review and editing. **Aalya Hamouda:** Writing—review and editing. **Anouk Leathead:** Writing—review and editing. **Ilan Ohana:** Writing—review and editing. **Rosie Foucault:** Writing—review and editing. **Iman Sadri:** Writing—review and editing. **Jeffrey A. Sioufi:** Writing—review and editing. **Nick Lee:** Writing—review and editing. **Tarek Benzouak:** Writing—review and editing. **Liam Murad:** Writing—review and editing. **Nicholas J. Corsi:** Writing—review and editing. **John Klein:** Data collection or management. **Inderjit Singh:** Data collection or management. **James Kearns:** Data collection or management. **Cecilia Chang:** Formal analysis; visualization. **Juan Justo Quintas:** Data collection or management; protocol/project development. **Kevin C. Zorn:** Conceptualization; supervision; writing—review and editing; data collection or management; protocol/project development. **Tiago Rodrigues:** Data collection or management; protocol/project development. **Shawn H. Marhamati:** Writing—review and editing; data collection or management; protocol/project development. **Brian T. Helfand:** Writing—review and editing; data collection or management; protocol/project development. **Alexander P. Glaser:** Project administration; supervision; conceptualization; writing—review and editing; data collection or management; protocol/project development.

## CONFLICT OF INTEREST STATEMENT

Alexander Glaser reports a relationship with PROCEPT BioRobotics Corporation that includes consulting or advisory, speaking and lecture fees, and travel reimbursement. Alexander Glaser reports a relationship with Emano Metrics that includes board membership and equity or stocks. Inderjit Singh reports a relationship with PROCEPT BioRobotics Corporation that includes consulting or advisory. James Kearns reports a relationship with PROCEPT BioRobotics Corporation that includes consulting or advisory, speaking and lecture fees, and travel reimbursement. Justo Quintas reports a relationship with PROCEPT BioRobotics Corporation that includes consulting or advisory. Kevin Zorn reports a relationship with PROCEPT BioRobotics Corporation that includes consulting or advisory, speaking and lecture fees, and travel reimbursement. Shawn Marhamati reports a relationship with PROCEPT BioRobotics Corporation that includes consulting or advisory. Brian Helfand reports a relationship with PROCEPT BioRobotics Corporation that includes consulting or advisory, speaking and lecture fees, and travel reimbursement. Other authors declare that they have no known competing financial interests or personal relationships that could have appeared to influence the work reported in this paper.
